# AbLang: an antibody language model for completing antibody sequences

**DOI:** 10.1093/bioadv/vbac046

**Published:** 2022-06-17

**Authors:** Tobias H Olsen, Iain H Moal, Charlotte M Deane

**Affiliations:** Department of Statistics, University of Oxford, Oxford OX1 3LB, UK; GSK Medicines Research Centre, GlaxoSmithKline, Stevenage SG1 2NY, UK; Department of Statistics, University of Oxford, Oxford OX1 3LB, UK

## Abstract

**Motivation:**

General protein language models have been shown to summarize the semantics of protein sequences into representations that are useful for state-of-the-art predictive methods. However, for antibody specific problems, such as restoring residues lost due to sequencing errors, a model trained solely on antibodies may be more powerful. Antibodies are one of the few protein types where the volume of sequence data needed for such language models is available, e.g. in the Observed Antibody Space (OAS) database.

**Results:**

Here, we introduce AbLang, a language model trained on the antibody sequences in the OAS database. We demonstrate the power of AbLang by using it to restore missing residues in antibody sequence data, a key issue with B-cell receptor repertoire sequencing, e.g. over 40% of OAS sequences are missing the first 15 amino acids. AbLang restores the missing residues of antibody sequences better than using IMGT germlines or the general protein language model ESM-1b. Further, AbLang does not require knowledge of the germline of the antibody and is seven times faster than ESM-1b.

**Availability and implementation:**

AbLang is a python package available at https://github.com/oxpig/AbLang.

**Supplementary information:**

Supplementary data are available at *Bioinformatics Advances* online.

## 1 Introduction

Recent progress within protein informatics has led to the development of pre-trained protein representations, derived from protein language models such as ESM-1b (Rives *et al.*, 2021), which have been used to perform state-of-the-art predictive tasks. Such protein language models require vast amounts of training data and so far have tended to use all protein sequences and therefore be general protein representations (Alley *et al.*, 2019; Elnaggar *et al.*, 2021; Rives *et al.*, 2021). With the creation of the Observed Antibody Space (OAS) database (Kovaltsuk *et al.*, 2018) and subsequent update (Olsen *et al.*, 2022), enough curated antibody sequences are now available to train a language model specifically for antibodies. An antibody specific model that has learnt the semantics of their sequences would allow for more precise predictions of antibody properties and new use cases.

Over the last decade, billions of antibodies have been sequenced (Chaudhary and Wesemann, 2018). However, in some cases, the sequenced antibodies are missing residues due either to sequencing errors, such as ambiguous bases (Huse *et al.*, 2007), or the limitations of the sequencing techniques used (Kim and Park, 2019). We find in OAS that ∼80% of the sequences are missing more than one residue at the N-terminus and ∼43% are missing the first 15 positions, and ∼1% contain at least one ambiguous residue somewhere in the sequence. The ability to accurately restore these missing residues would increase data availability and be of benefit to antibody drug discovery. Currently, sequence imputation can only be done by identifying the correct ImMunoGeneTics (IMGT) germlines from the IMGT/GENE-DB (Giudicelli *et al.*, 2005) and using the germline sequence to add the missing residues. This approach requires correctly determining the allele of the sequence, a process that can be time consuming and/or produce ambiguous results.

Here, we present AbLang, an antibody specific language model trained on either the heavy or light chain antibody sequences from OAS. While AbLang can be used to create representations for residue or sequence specific predictions and residue engineering, in this paper, we focus on showing how AbLang can be used to restore missing residues in antibody sequences, more accurately than using IMGT germlines or a general protein model like ESM-1b.

## 2 Materials and methods

**Fig. 1.**
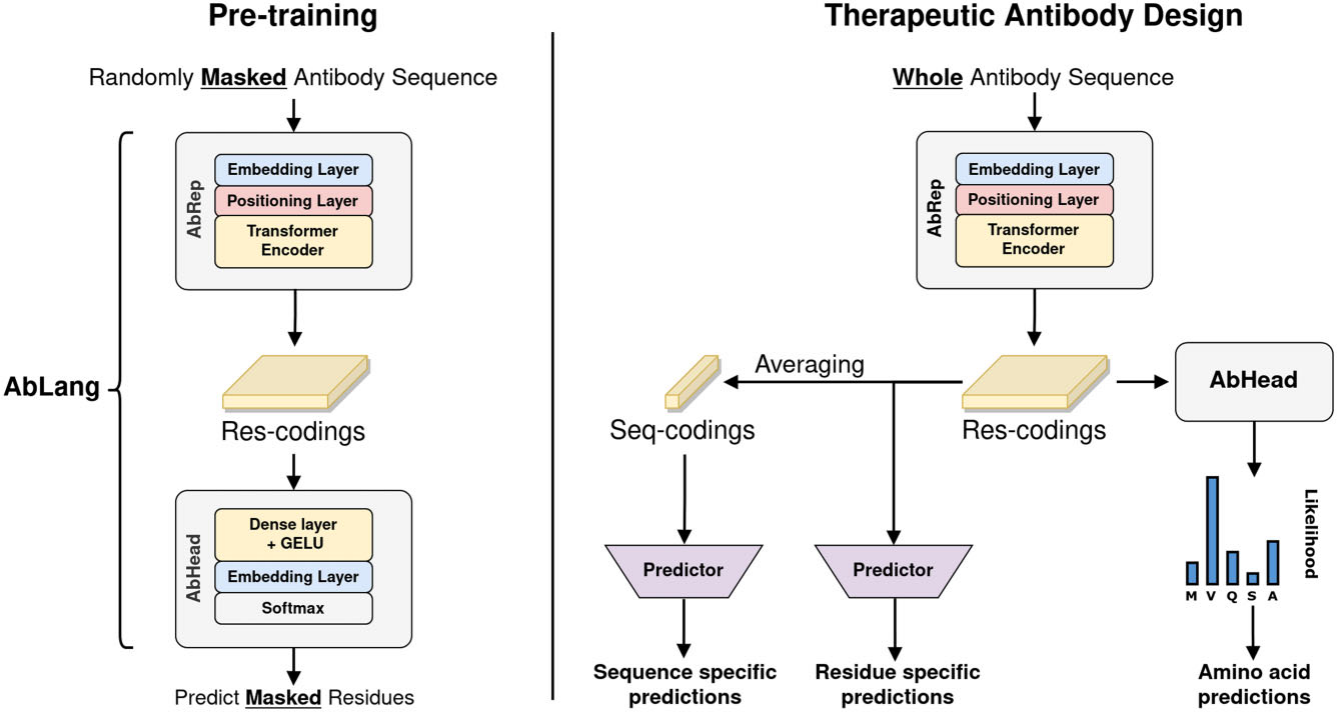
Overview of the architecture of AbLang, AbRep and AbHead, and examples of possible use cases. For pre-training, residues are randomly masked in each sequence and the masked residues are predicted and compared to the original residue. After pre-training, the model can, through different use cases, be used to improve therapeutic antibody design. AbHead can be removed and the res-codings from AbRep used for either residue or sequence specific predictions, or AbHead can be kept and used for restoring missing residues or exploring possible mutations

Two AbLang models were trained, one for heavy and one for light chains. Each AbLang model consists of two parts: AbRep, which creates representations from antibody sequences, and AbHead, which uses the representations to predict the likelihood of each amino acid at each position (Fig. 1).

AbLang was implemented using PyTorch 1.8.1 and was inspired by HuggingFace's (Wolf, 2020) Transformer 3.0.2 library. AbRep follows the architecture of RoBERTa (Liu *et al.*, 2019), except it uses a learned positional embedding layer with a max length of 160. Each of its 12 transformer blocks has 12 attenuated heads, an inner hidden size of 3072 and a hidden size of 768. From AbRep, the res-codings (768 values for each residue) are obtained. AbHead follows the design of RoBERTa's head model, with a hidden size of 768.

During training, between 1% and 25% of residues from each sequence were selected, and of these, 80% were masked, 10% randomly changed to another residue and 10% left unchanged. One AbLang model was trained on heavy chain sequences for 20 epochs with a batch size of 8192, and another on light chain sequences for 40 epochs with a batch size of 4096. Both models were optimized using an Adam optimizer with a linear warm-up period for 5% of the steps, a peak learning rate of 0.0002, a learning rate decrease following a cosine function, and a weight decay of 0.01. For every dropout and layer normalization, a 0.1 rate and 1e^−^^12^ epsilon was used. The hyperparameters were selected to be similar to those used in the RoBERTa paper (Liu *et al.*, 2019).

## 3 Results

### 3.1 Data preparation

All antibody sequences seen three or more times in the OAS database as of October 2021 were downloaded. The heavy and light chain sequences were then clustered separately based on identical CDR3s and thereafter clustered further by 70% identity over the whole sequence using Linclust (Steinegger and Söding, 2018), with the longest sequence selected from each cluster. The selected sequences were then randomly divided into training sets of 14 126 724 heavy and 187 068 light sequences, and two evaluation sets of 100 000 heavy and 50 000 light sequences. The training sets were then used to train AbLang as described in Section 2.

### 3.2 Ablang's antibody sequence representations

AbLang can be used to generate three different sets of antibody sequence representations. The first representation, the res-codings, consists of 768 values for each residue, useful for residue specific predictions. The second representation, the seq-codings, represents the whole sequence and is derived from the mean of all res-codings in a sequence. The seq-codings are 768 values for each sequence and are useful for sequence specific predictions. Additionally, they have the benefit of having the same length for each sequence, removing the need to align antibody sequences. Lastly, AbLang can be used to generate the likelihoods of each amino acid at each position in a given antibody sequence, useful for antibody engineering.

**Fig. 2.**
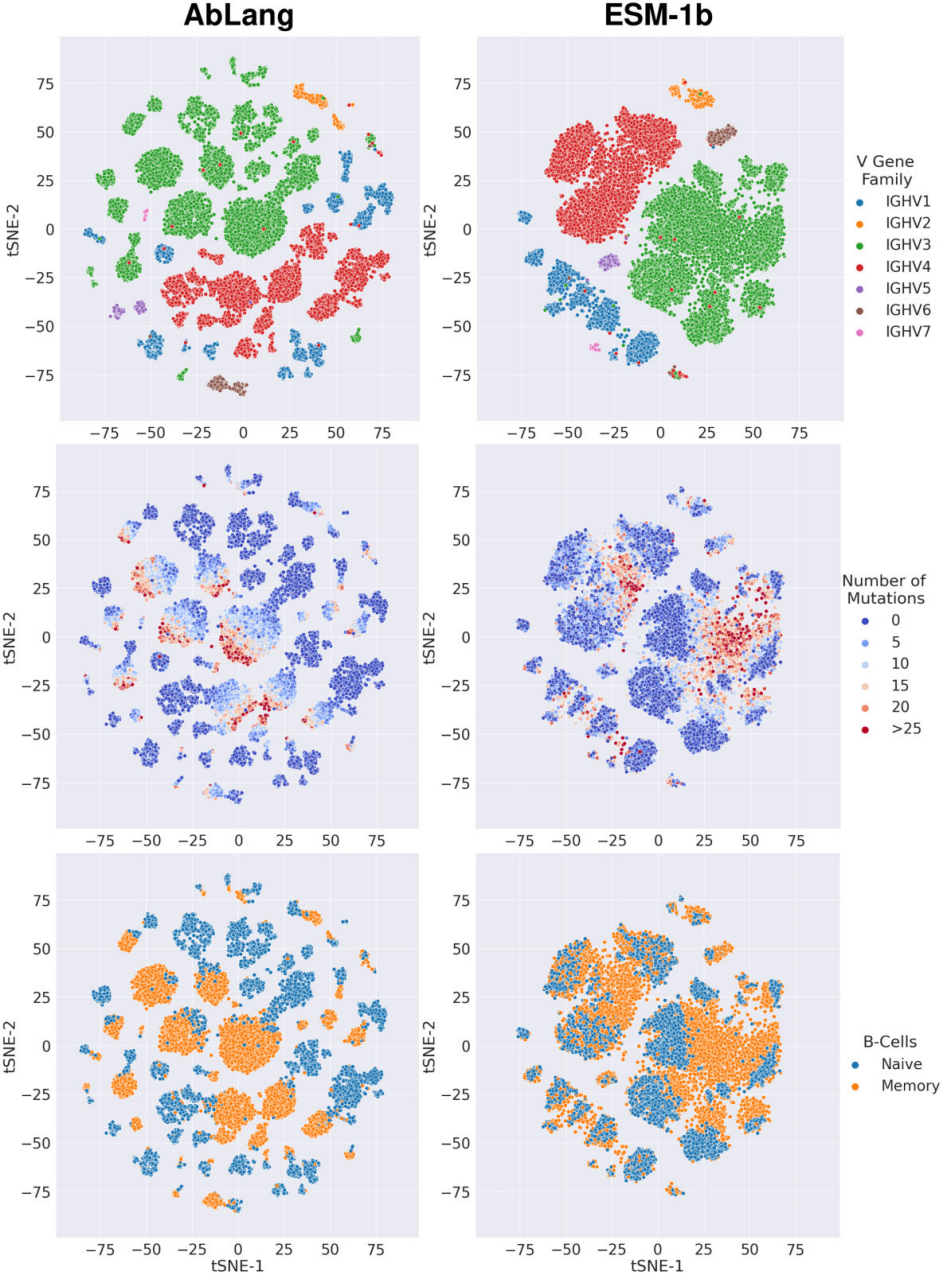
Comparison of AbLang and ESM-1b representations at clustering sequences based on their V-genes, originating cell type and number of mutations

To investigate the sequence information extracted by AbLang and compare it to that of ESM-1b, we visualized the AbLang and ESM-1b sequence representations of 10 000 naïve and 10 000 memory B-cell sequences from Ghraichy *et al.* (2021) with a t-SNE (van der Maaten and Hinton, 2008) plot (Fig. 2).

As Figure 2 shows, AbLang and ESM-1b both separate the sequences based on their V-gene families; however, AbLang separates the V-genes into smaller clusters. These smaller clusters can partly be attributed to AbLang's finer separation of V-genes (see Supplementary Fig. S1). Within the AbLang clusters, a clearer separation can be seen between naive B-cells and memory B-cells than with ESM-1b's clusters. Further, the memory B-cells, in AbLang's clusters, appear to be ordered based on a gradual increase in mutations. This potentially indicates that AbLang representations contain information about the order of antibody mutations.

### 3.3 Ablang for restoring missing residues

**Fig. 3.**
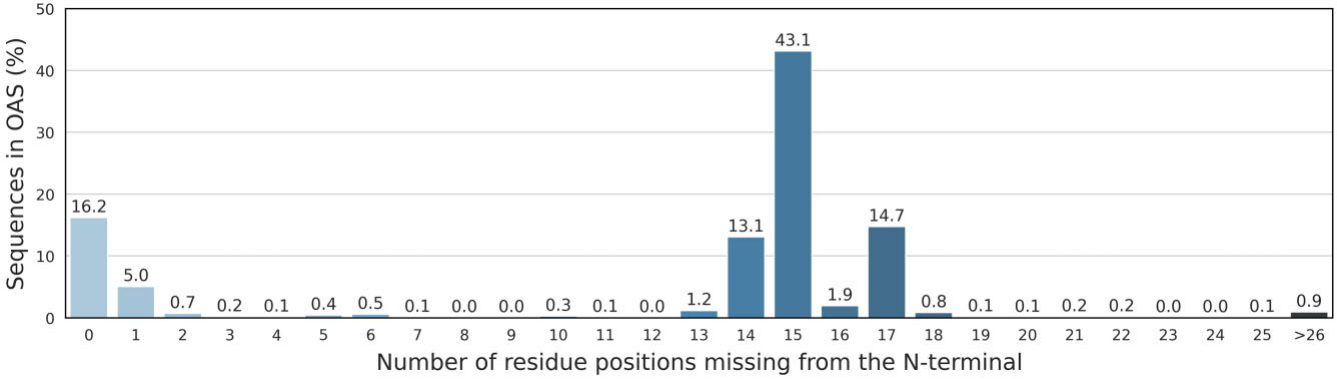
Overview of the antibody sequences in OAS, showing the percentage of sequences and number of residues they are missing from the N-terminus. Over 40% of the sequences in OAS are missing the first 15 residues

AbLang's representations can be used for a plethora of antibody design applications. As an example, we use AbLang to restore missing residues in antibody sequences. Figure 3 demonstrates the need for such a tool, showing how over 40% of the sequences in OAS are missing the first 15 residues and ∼80% of the sequences are missing more than one residue at the N-terminus.

**Fig. 4.**
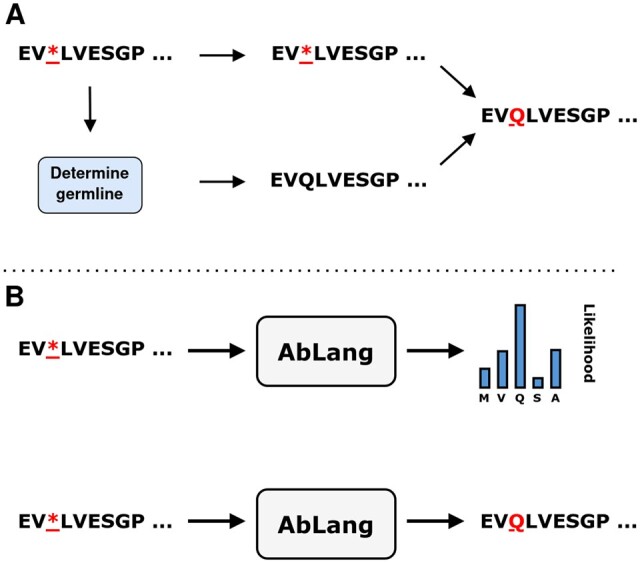
Illustration of how the IMGT germline and AbLang method restores missing residues. (**A**) From an input sequence, the germline is determined and then used to restore missing residues. (**B**) An input sequence has asterisks at the positions to predict. AbLang predicts the amino acid with the highest likelihood

The input to AbLang for sequence restoration is an antibody sequence with asterisks for unknown residues (Fig. 4). AbLang restores the missing residues by predicting the likelihood of each amino acid at the marked positions, with the amino acid with the highest likelihood then selected as the prediction.

We tested the ability of AbLang to restore both the N-terminus of antibody sequences and missing residues randomly scattered throughout the sequence. From the evaluation sets, 100 complete sequences for each of the 20 heavy and 42 light human V alleles seen in the evaluation set were randomly selected. These 2000 heavy and 4200 light sequences were used as the test set.

**Fig. 5.**
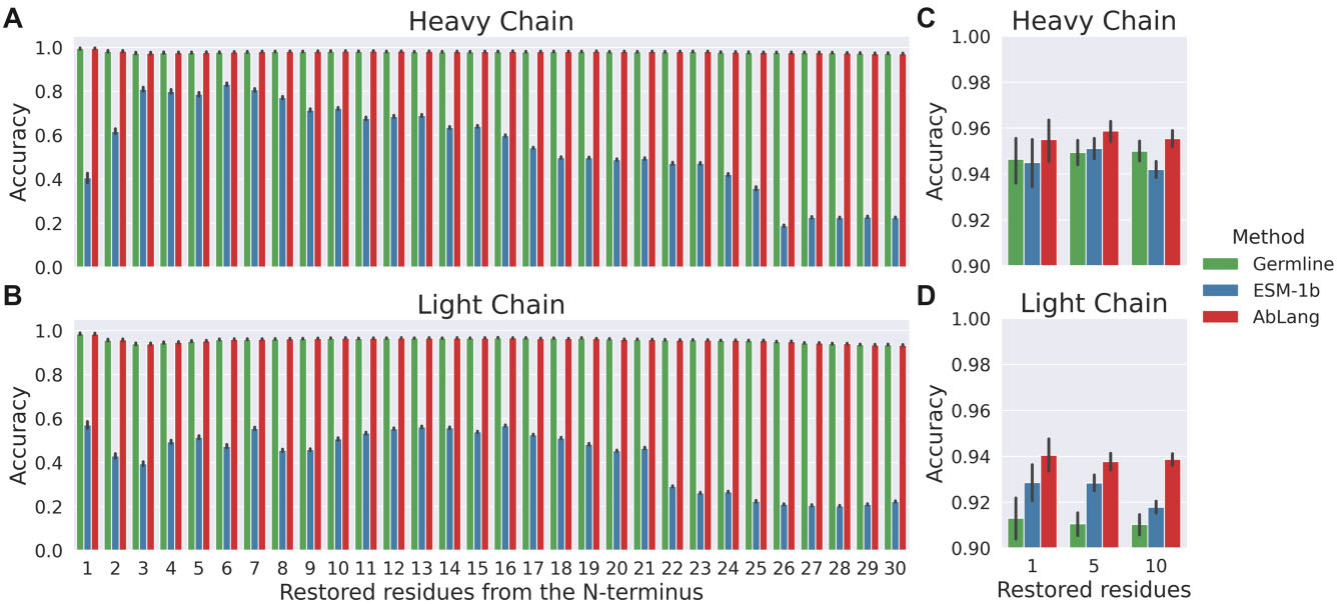
Antibody sequence restoration using IMGT germline sequences, a general protein language model ESM-1b and the antibody specific language model AbLang. (**A** and **B**) The restoration of sequences missing up to 30 residues of the N-terminus, and (**C** and **D**) the restoration of sequences with a random set (1, 5 or 10) of missing residues

Figure 5 shows a comparison of AbLang, to the general protein model ESM-1b and to the use of germline residues for the prediction of missing residues in an antibody sequence. Sequences were numbered in the IMGT scheme using ANARCI (Dunbar and Deane, 2016) and positions from 1 up to 30 were masked and then restored using the three different methods. The accuracy of this restoration was measured as the percentage of correctly predicted amino acids. IMGT germlines and AbLang achieve comparable accuracy, both restore missing N-terminus residues with accuracies of around 96% and 98% for the first 15 positions of the light and heavy chain, respectively. ESM-1b has far poorer performance achieving accuracies of 54% and 64%. The performance of IMGT germlines and AbLang is very similar, but the IMGT germline method requires knowledge of or accurate prediction of the germline, while AbLang can be rapidly deployed without any additional information.

In some cases, sequencing errors can result in residues being unknown at random sites throughout the antibody sequence. The ability of AbLang, IMGT germlines and ESM-1b to predict residues at randomly selected positions was also compared. Using the same test set as above, 1, 5 or 10 residues were randomly masked in each sequence's V-region. AbLang is more accurate at this task than both IMGT germlines and ESM-1b for both heavy and light chains. AbLang is also the fastest of the three methods, able to process 100 sequences in 6.5 s to ESM-1b's 44.9 s, using 4 cores on an Intel Core i7-10700.

Often the number of missing residues at the N-terminus is unknown. To overcome this problem, we tested the use of ANARCI numberings and AbLang's predicted likelihood of the first residue together to determine the correct number of missing residues.

The ANARCI numbering of antibody sequences gives an initial reasonable approximation of the number of residues missing from the N-terminus. However, because of possible indels and the variable length of CDR1, the ANARCI numbering alone is unable to determine the correct number of residues missing from the N-terminus. We observed that AbLang's predicted likelihood of the first residue in a sequence, was a good approximation of whether a sequence is the whole variable region. We therefore used the likelihood of the first residue to identify if a sequence has been restored with the correct number of residues at its N-terminus.

We tested N-terminus lengths between eight residues shorter and up to two residues longer than the standard length given by ANARCI. This takes into account possible indels and a CDR1 region containing 5–12 residues. This process can be repeated and we found that this often improves the results, especially for heavy chains.

**Fig. 6.**
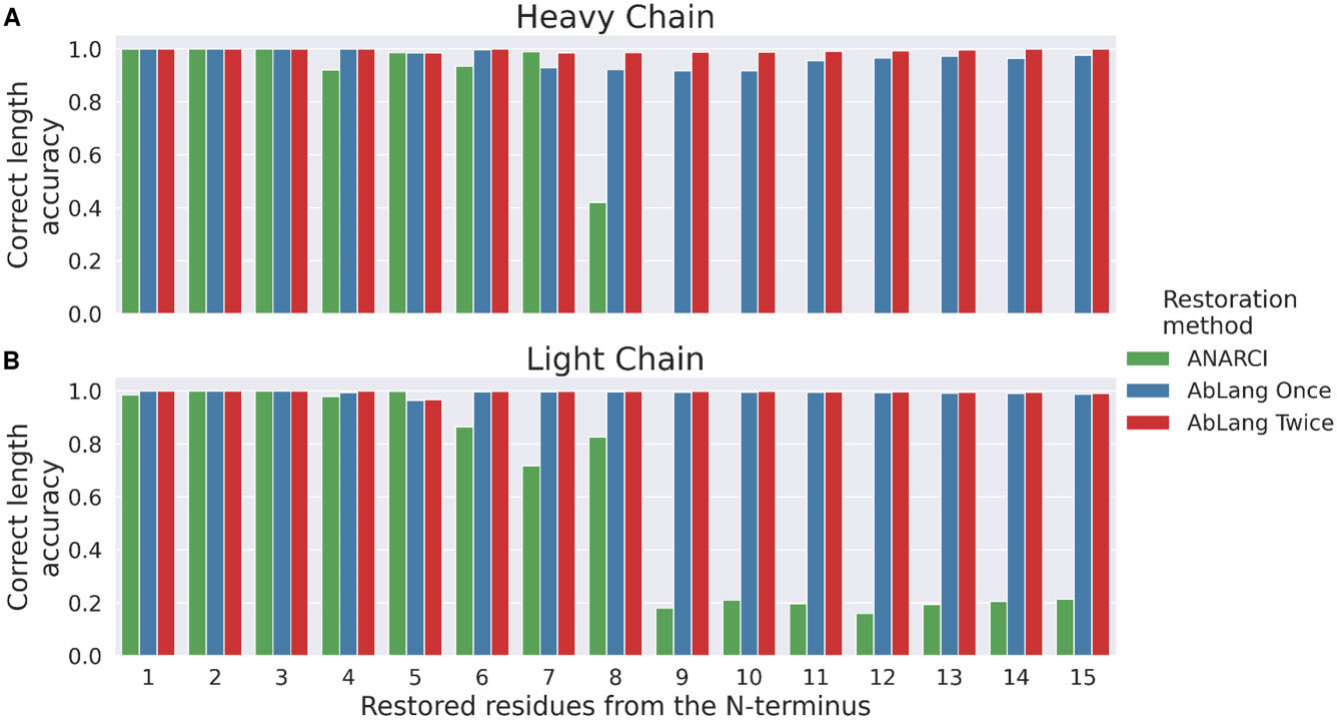
Comparison of the restoration of antibody sequences missing unknown numbers of residues at the N-terminus, using the standard length given by ANARCI or the AbLang predicted likelihood of the first residue to determine the correct length. With AbLang, sequences were restored once or twice. The accuracy of selecting the correct number of residues at the N-terminus to restore for light (**A**) and heavy (**B**) sequences missing up to 30 residues of the N-terminus is shown

Figure 6 compares the standard length given by ANARCI (green) with the ability of AbLang to restore the correct number of missing N-terminus residues, by either restoring once (blue) or twice (red). If the first 15 positions are missing, the ANARCI given length is correct for only one heavy chain sequence and 21.3% of the light chains, while restoring once with AbLang leads to the correct number of missing residues for 98.7% and 97.6% of the light and heavy chains, respectively. For improved performance, the restored sequences can go through the process again. This increases the restoration of the correct number of missing residues to 99.1% and 99.9% for light and heavy chains, respectively. ANARCI's inability to account for indels, such as the common deletion at position 10, can be seen in Figure 6, where the ANARCI given length is highly inaccurate when nine or more residues are missing.

## 4 Discussion

A language model specific for antibodies, should learn a deeper understanding of the semantics of antibodies than current general protein language models. In this work, we present AbLang, a language model trained on a large dataset of antibody sequences from OAS.

AbLang can be used to derive three different sets of antibody representations, each with different possible use cases, as illustrated in Figure 1. Res-codings could be used for residue specific representations or predictions and seq-codings for sequence specific representations or predictions, and the likelihoods from AbHead could be used to predict amino acids at each position in a given antibody sequence. All three sets of representations are easily obtainable using the freely available AbLang python package at https://github.com/oxpig/AbLang.

To demonstrate that AbLang has learnt a useful sequence representation of antibodies, we show how AbLang's seq-codings contain knowledge of the germlines, originating cell type and number of mutations (see Fig. 2). However, these t-SNE visualizations are only indicative, and future work could explore these observations further.

To showcase AbLang's usefulness for antibody design, we explored its ability to restore missing residues in an antibody sequence. As shown in Figure 3, for 80% of available antibody sequence data at least one residue is missing from the N-terminus. Sequences with missing residues are usually discarded, significantly diminishing available data. Accurate restoration of the N-terminus therefore allows the available data for further analysis to be more than doubled.

We demonstrate the use of AbLang to restore missing residues in antibody sequences, and show how AbLang performs on par or better than using IMGT germlines, but without the need to have knowledge of the germline. Further, we describe a method for using AbLang to restore N-terminus regions with unknown arbitrary lengths.

The baseline IMGT germline method represents predicting the unmutated sequence. A better accuracy than this method therefore implies predictions which are not just the most often seen amino acid at a position and instead are specific to the input sequence. Further, we show how AbLang restores residues more accurately and faster than a current state-of-the-art protein language model ESM-1b, emphasizing the benefits and potential of an antibody specific language model.

Overall the work shows the possibility of using protein language models for restoring residues in protein sequences, a crucial problem not only for antibody sequences but also for protein sequences in general. Though ESM-1b struggles with restoring longer end regions, it outperforms the IMGT germline baseline when restoring randomly placed residues in antibody sequences. ESM-1b might therefore be a useful tool for restoring a few missing residues in proteins, but less useful at restoring the ends of sequences and longer regions. The fact that ESM-1b struggles to restore residues at the N-terminus compared to single randomly distributed residues, could be because longer regions give rise to higher combinations of possible residues, and as ESM-1b does not have antibody specific context, it is unable to make accurate predictions.

In this work, we give an example of how our antibody specific language model AbLang, can be used to create state-of-the-art solutions for antibody design. However, AbLang could be used for a wide range of other antibody discovery and design problems, which we hope by making it available, we and others can explore in future work.

## Supplementary Material

vbac046_Supplementary_Data

## Data Availability

The data underlying this article are available in the Oberserved Antibody Space (OAS) at http://opig.stats.ox.ac.uk/webapps/oas/.
