# Supplementary material for: AbLang: an antibody language model for completing antibody sequences
Source: Bioinform Adv. 2022 Jun 17;2(1):vbac046. doi: 10.1093/bioadv/vbac046 (PMC9710568; doi:10.1093/bioadv/vbac046)
Supplement: vbac046_Supplementary_Data [file vbac046_supplementary_data.pdf]

Supporting Information for

AbLang: An antibody language model for completing antibody sequences

Tobias H. Olsen[1], Iain H. Moal[2] and Charlotte M. Deane[1]

[1] Department of Statistics, University of Oxford, Oxford OX1 3LB, United Kingdom
[2] GSK Medicines Research Centre, GSK, Stevenage SG1 2NY, United Kingdom

Corresponding: deane@stats.ox.ac.uk

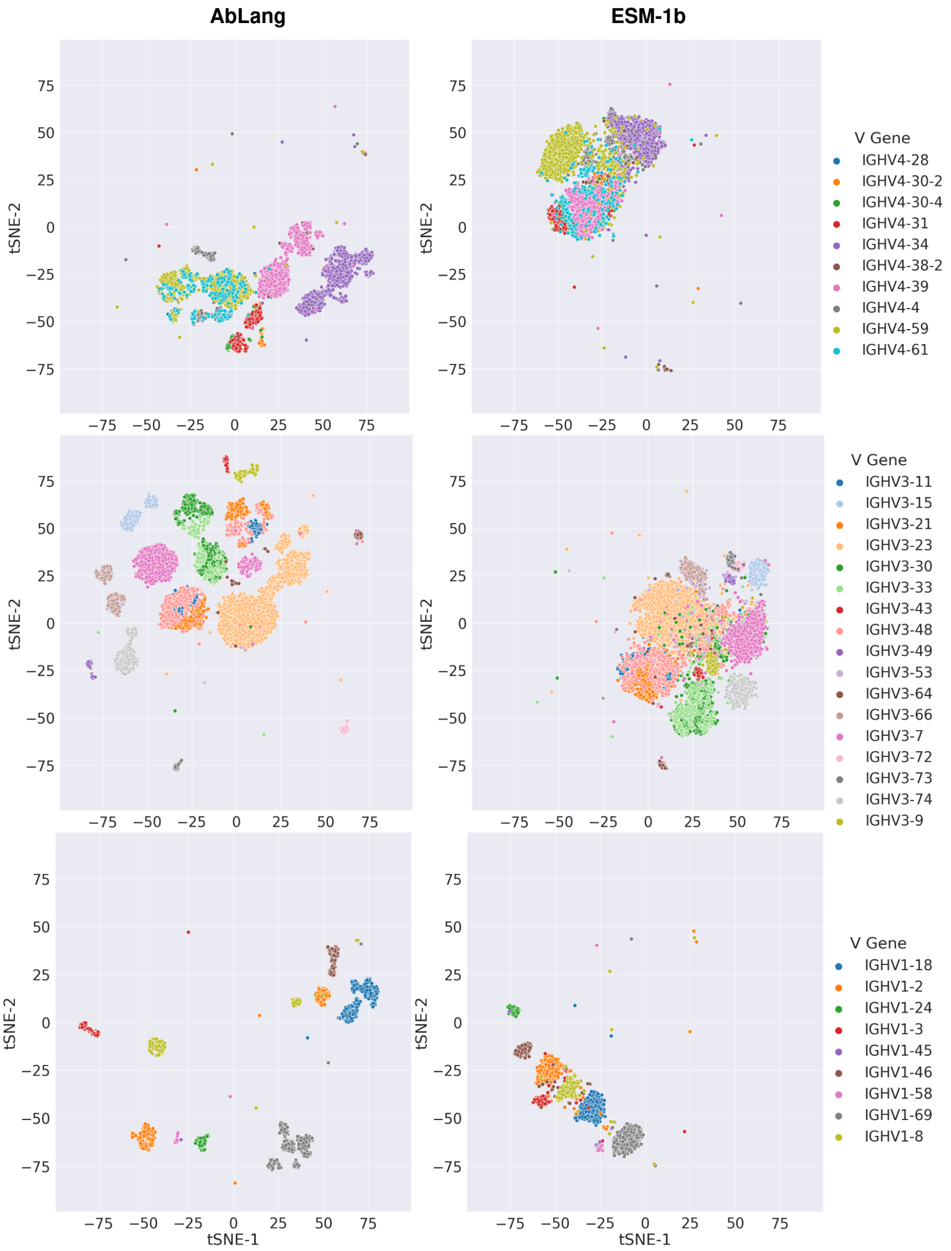

Figure S1: Comparison of AbLang and ESM-1b representations at clustering sequences based on their V-genes. The figure compares the three most common heavy chain V-gene families in our dataset, IGHV1, IGHV3 and IGHV4.
